# Combination of nucleic acid amplification and CRISPR/Cas technology in pathogen detection

**DOI:** 10.3389/fmicb.2024.1355234

**Published:** 2024-02-06

**Authors:** Dandan Zeng, Jinlong Jiao, Tianlu Mo

**Affiliations:** School of Health Science and Engineering, University of Shanghai for Science and Technology, Shanghai, China

**Keywords:** nucleic acid amplification, CRISPR/Cas, rapid detection, pathogen detection, specific

## Abstract

Major health events caused by pathogenic microorganisms are increasing, seriously jeopardizing human lives. Currently PCR and ITA are widely used for rapid testing in food, medicine, industry and agriculture. However, due to the non-specificity of the amplification process, researchers have proposed the combination of nucleic acid amplification technology with the novel technology CRISPR for detection, which improves the specificity and credibility of results. This paper summarizes the research progress of nucleic acid amplification technology in conjunction with CRISPR/Cas technology for the detection of pathogens, which provides a reference and theoretical basis for the subsequent application of nucleic acid amplification technology in the field of pathogen detection.

## Graphical abstract

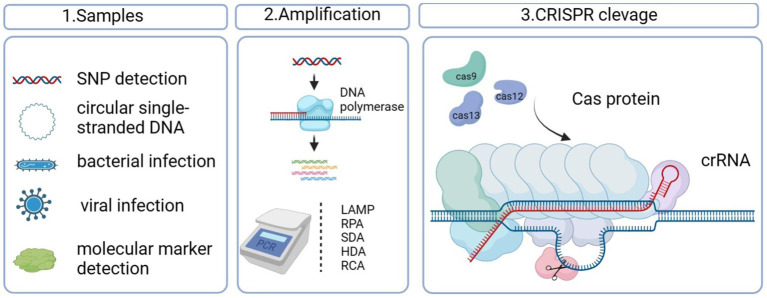

## 1 Introduction

A pathogen is defined as an infectious micro-organism or agent, of which viruses and bacteria are the most encountered clinically (Casadevall and Pirofski, 2002). These pathogens are highly evolvable, pathogenic and rapidly spreading, posing a serious threat to human health. Microbial control programmes are increasingly used throughout society to reduce the risk of consumer infection. The bacterial culture method is widely recognised as the “gold standard” for pathogen detection due to its robustness in common laboratory experiments. However, it has disadvantages such as time-consuming, laborious, and inefficient detection, which have significantly hindered its widespread use in the clinic. An alternative method is immunological detection, which is based on the recognition and binding of antigens by specific antibodies (Kohl and Ascoli, 2017). Although it is advantageous in terms of speed, simplicity and specificity in the detection of pathogenic microorganisms, it requires a long period of time for antibody preparation and has a rather low detection sensitivity. Nucleic acid detection technologies, unlike the above-mentioned methods, can simultaneously meet the requirements of accuracy, rapidity and sensitivity for pathogen detection, thus showing superiority in ensuring human safety.

Nucleic acid amplification tests (NAATs) are the most widely used nucleic acid detection technology and have become indispensable tools throughout the life sciences since polymerase chain reaction (PCR) became widely used in biomedicine and other fields in 1980 (Seemayer, 1990). This paper mainly introduces the application of nucleic acid amplification technology combined with the Clustered Regularly Interspaced Short Prepeats (CRISPR)-Cas system in the field of pathogen detection, which provides a reference for the subsequent application of nucleic acid amplification technology in the field of rapid detection.

## 2 Detection of pathogens based on PCR-CRISPR

PCR is the most widely used technical tool for pathogen detection based on nucleic acid amplification. Simulating the process of DNA replication in the central dogma, the PCR process works *in vitro* by replicating the daughter strand of DNA that is complementary to the parent strand of template DNA after a cycle of denaturation-annealing-extension (Ramesh et al., 1992; Markoulatos et al., 2002). Specific primers are designed to detect different targets, and the target DNA amplification products accumulate exponentially within 25–30 PCR cycles. Due in part to its “amplification” nature, PCR has become a multifunctional tool with improved specificity and sensitivity compared to immunological assays, and can be used to detect a variety of pathogenic bacteria (Malorny et al., 2003; Hadi et al., 2023). To improve the detection performance of PCR, multiplex PCR (mPCR) and quantitative real-time PCR (qPCR) have also been established with the advancement of molecular biology and PCR technology. Alnakip et al. (2023) characterized *Staphylococcus aureus* and *Escherichia coli* in 150 cheese samples by multiplex PCR to evaluate their ability to produce virulence factors. Anejo-Okopi et al. (2016) designed specific primers to detect *Salmonella* virulence gene invA from selected poultry farms using PCR with 91% success rate. Amplification of virulence genes of suspected *Salmonella* in poultry using PCR is effective and can be used as an alternative tool for rapid detection of *Salmonella*. However, because the display of the detection results of the above methods is typically based on nucleic acid electrophoresis in either ultraviolet or fluorescence mode, these PCR-only detection methods have drawbacks such as long detection time and inconvenience of on-site testing. How to read the PCR results quickly and easily has become a practical problem that needs to be solved.

**Figure 1:**
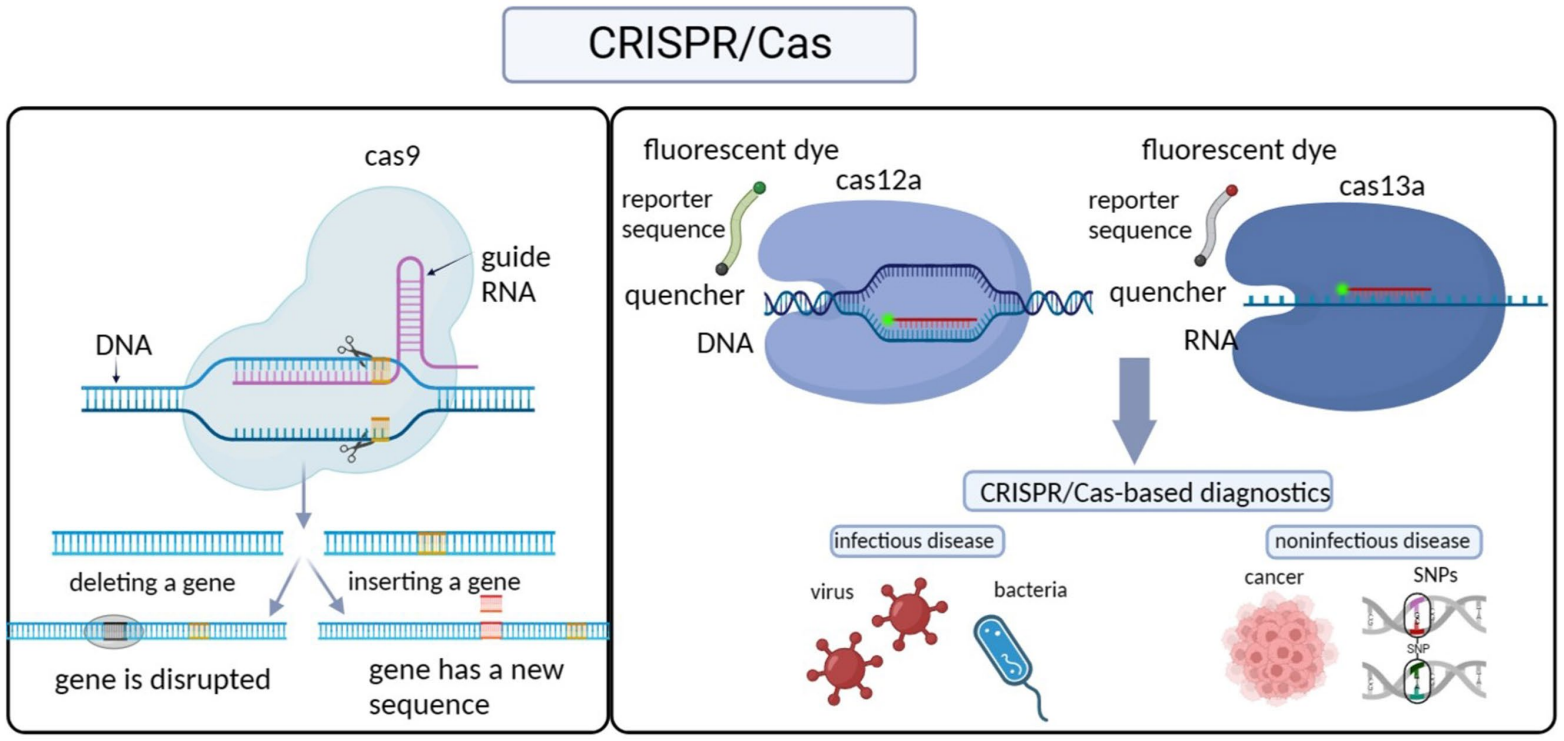
Illustration of CRISPR-Cas sensing mechanisms and their diagnostic application.

As part of the acquired immune system in prokaryotes, the CRISPR/Cas system defends the host organism against the invasion of exogenous genetic elements by destroying foreign plasmid or phage DNA, and this process is centered on CRISPR sequences and Cas proteins (Koonin and Wolf, 2015; Hryhorowicz et al., 2017; Paul and Montoya, 2020). Specifically, the CRISPR sequence is first transcribed and processed into a short crRNA that directs the recognition of the target DNA. The Cas proteins, known as nucleic acid endonucleases, then bind the crRNA to form an RNA-protein complex. After further binding to the target DNA under the guidance of the crRNA, the Cas proteins then cut the target DNA, creating a gap in the gene. Gene repair by homologous recombination or non-homologous end joining enables gene editing (Figure 1; Savić and Schwank, 2016; Paul and Montoya, 2020; Liu et al., 2022).

**Figure 2:**
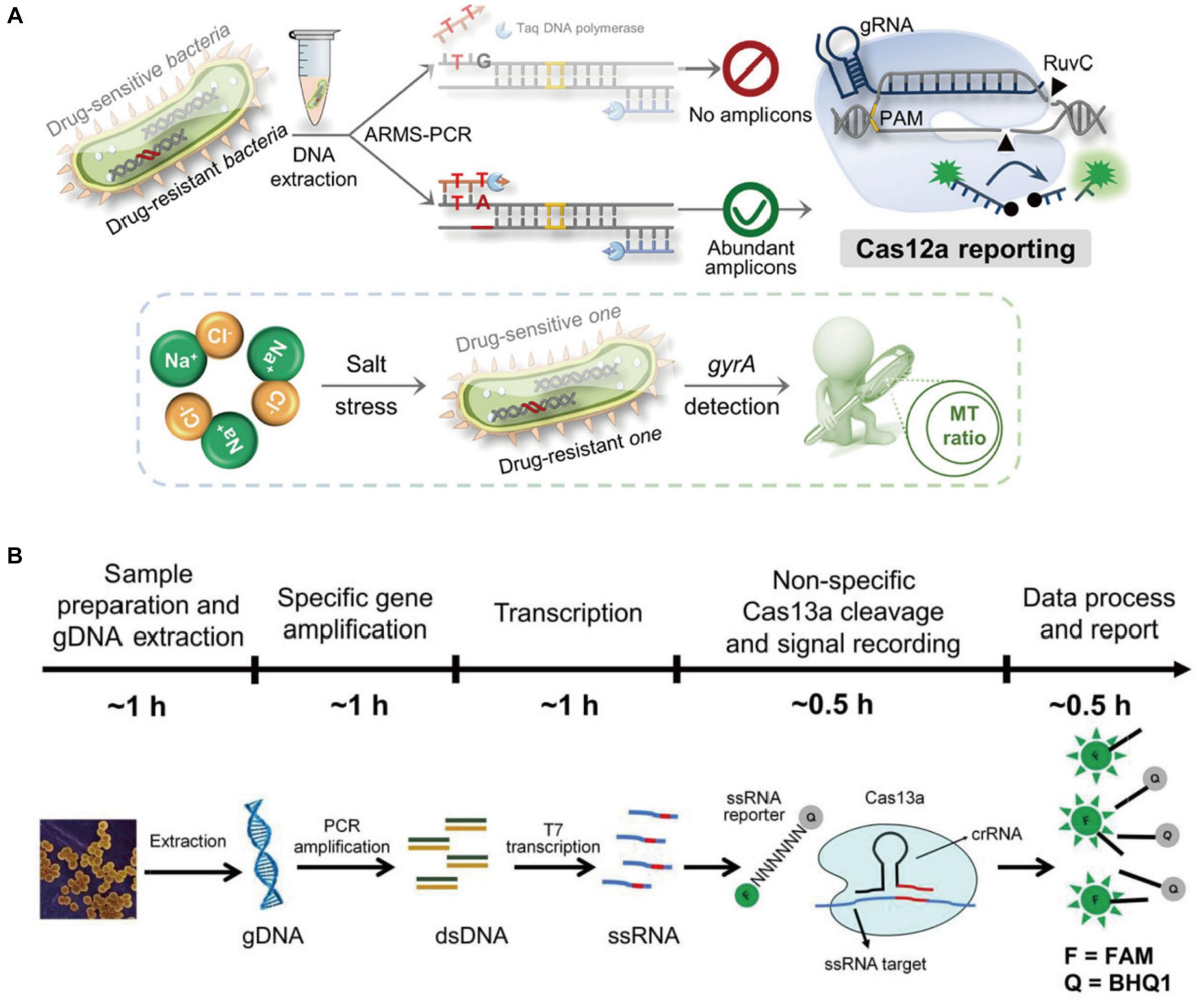
**(A)** Schematic illustration of the Carms assay for distinguishing *Salmonella enterica* species with single-nucleotide resolution and the investigation of the resistance of drug-resistant *S. enterica* to salt stress (Yang et al., 2022); **(B)** proposed CCB-Detection for *Staphylococcus aureus* sensing (Zhou et al., 2020).

In a study conducted by Wang et al. (2023) they developed a method that can detect the presence of terminators in transgenic plants using a combination of PCR and CRISPR/Cas12a techniques. The method can be used to identify the target gene levels in about 50 minutes. In addition, accurate detection of all 11 samples confirmed the applicability. Yang et al. (2022) integrated the CRISPR/Cas system to amplify the amplification refractory mutation system qPCR (ARMS-qPCR) to detect single nucleotide polymorphism (SNP) in *Salmonella enterica*. The results of the experiment revealed that the detection rate was 0.5%, which is lower than the limit of detection that gel electrophoresis can provide. This method can be utilized to analyze the various genes that are involved in the resistance to drugs (Figure 2A). Cas13a, a remarkable Cas protein, exhibits non-canonical RNAase activity that can be activated by crRNA-mediated target RNA recognition. This feature allows the combinatorial use of Cas13a activity and isothermal amplification. Due to the programmability of the crRNA sequence and the spin-off effect of the Cas13a protein. Zhou et al. (2020) developed a CRISPR/Cas13a-based Bacterial Detection (CCB) assay to directly detect *Staphylococcus aureus* (*S. aureus*) after PCR amplification with high sensitivity and selectivity. The results showed that the CCB assay was able to successfully detect target genomic DNA (gDNA) down to 10^0^ Am with LOD of 1 cfu/mL, and the dynamic detection range of *S. aureus* was 10^0^ ~ 10^7^ cfu/mL (Figure 2B).

PCR, as the gold standard of molecular detection, is reproducible, sensitive and widely applicable, but requires electrophoresis, color development and other steps, which are instrument-dependent, complicated and not convenient for on-site detection. Despite the widespread application of PCR, amplifying complex or long DNA segments is challenging. The discovery of the CRISPR/Cas technology provides a feasible alternative to on-site detection of PCR technology. Meanwhile, the application of CRISPR/Cas system also provides new ideas and methods for the optimization and improvement of PCR technology. In order to overcome the shortcomings of PCR, researchers have explored new molecular detection techniques by combining PCR technology with CRISPR/Cas technology, and through further improvement and optimization, the technology has been widely used in many fields and has had a great impact (Rakshit et al., 2022; Zhang et al., 2022; Cao et al., 2023; Meng et al., 2023; Tian et al., 2023).

## 3 Pathogen detection based on isothermal nucleic acid amplification-CRISPR

Despite the flourishing development of PCR techniques and its widespread use in combination with the CRSPR/Cas technique for molecular detection, the need for a thermal cycling step during PCR experiments has significantly hindered its use for on-site real-time detection. Isothermal nucleic acid amplification, a constant-temperature nucleic acid amplification technique, has circumvented the need for the demanding thermal cycling step in a canonical PCR experiment, and therefore can usually complete the amplification process in a shorter time while maintaining high efficiency, sensitivity, and accuracy. Representative isothermal amplification technologies mainly include Loop-Mediated Isothermal Amplification (LAMP) (Notomi et al., 2000), Recombinase Polymerase Amplification (RPA) (Piepenburg et al., 2006), Strand Displacement Amplification (SDA) (Walker et al., 1992), Helicase Dependent Amplification (HDA) (Vincent et al., 2004), Rolling Circle Amplification (RCA) (Nilsson et al., 1994), Hybridization Chain Reaction (HCR) (Dirks and Pierce, 2004), etc. It is noteworthy that a canonical isothermal amplification process typically requires an indicator readout (e.g., fluorescent probes) to detect and report the amplification products, while the specificity of the detection process can often be affected by the sequence of the target itself and the specificity of the amplification process. Given the inherent limitations of isothermal amplification, the CRSPR/Cas detection system can serve as both a highly specific method for nucleic acid sequence identification and an idle readout of the amplification process, and the combined use of isothermal amplification and CRSPR/Cas techniques can significantly improve the accuracy of test results.

### 3.1 LAMP-CRIPSR

LAMP technology is a new thermostatic nucleic acid amplification technology suitable for genetic diagnosis reported by Notomi et al. (2000), which has been widely used in the rapid detection of a variety of pathogens. This technique relies on (1) the catalysis of Bst DNA polymerase with strand-substitution ability, and (2) the design of a pair of external primers (i.e., F3 and B3) and a pair of internal primers (i.e., FIP and BIP). The primers can bind to the target sequence under the condition of 60–65°C, and the product is the structure of stem-loop DNA, which can realize 10^9^–10^10^ times amplification within 15–60 min (Nagamine et al., 2001; Parida et al., 2008; Notomi et al., 2015; Becherer et al., 2020; Park, 2022). The amplification products can then be detected by gel electrophoresis or turbidimetry. Pyrophosphate byproducts are produced during the reaction due to the large amount of DNA being synthesized, and the compound binds to divalent metal ions to form insoluble compounds, which can be used to further observe the amplification reaction. This technology is not only able to use DNA as a template but also to detect target RNA by reverse transcription-LAMP (RT-LAMP) reaction. By using an AMV reverse transcriptase, it is also possible to complete the reaction in one step in a short period of time and under constant temperature conditions (Tomita et al., 2008).

**Figure 3:**
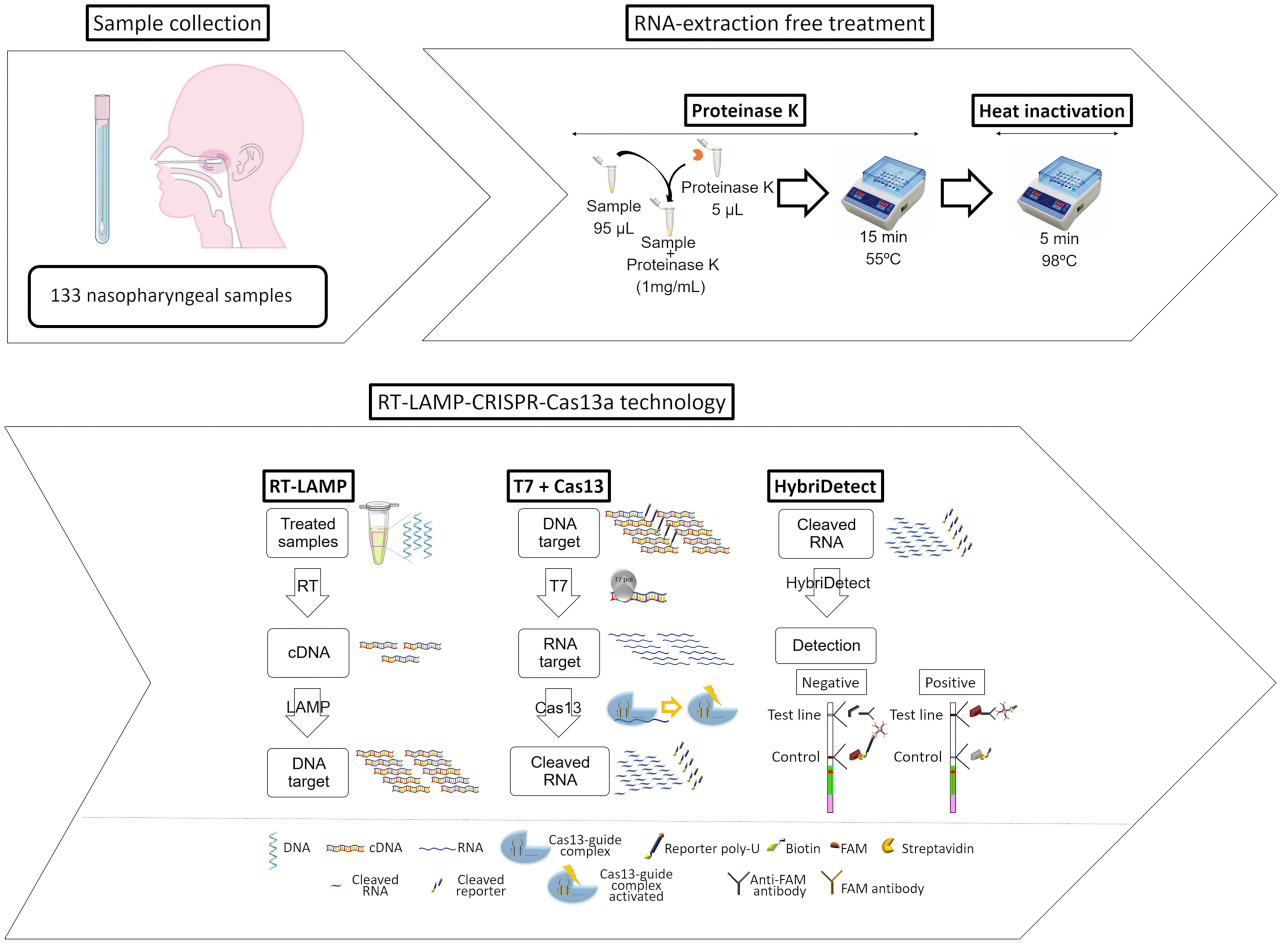
Workflow of the novel developed and optimized protocol for infectious disease diagnosis based on CRISPR-Cas13a technology (Ortiz-Cartagena et al., 2022).

Bao et al. (2020) reported a contamination-free LAMP assisted by CRISPR/Cas9 cleavage, which is superior to CRISPR/Cas9 in terms of reliability and durability. The core of the methodology is to design forward or reverse internal primers in target-independent regions so that the LAMP product contains an in-situ neighboring motif (PAM) site for CRISPR/Cas9 to recognize and cleave. A LAMP-CRISPR/Cas12a-based lateral flow biosensor was developed and used by Lee and Oh (2023) to detect *Salmonella* with a LOD of 1.22 × 10^0^ CFU/mL. Wang et al. (2021) combined RT-LAMP with Cas12a to detect SARS-CoV-2, and a single-molecule sample could be detected in 45 min, which was consistent with the results of RT-PCR, and this method has the characteristics of short detection time, high sensitivity, and visualization of results. The paper equipment based on RT-LAMP and Cas12a by Cao et al. (2022) developed an RT-LAMP with Cas12a based paper device for SARS-CoV-2 detection in wastewater, where enriched wastewater samples were lysed and introduced into a paper device for detection of specific gene fragments, and the results could be read at 480 nm or in a paper device. Li et al. (2022) investigated the establishment of Immunocapture Magnetic Bead (ICB) enhanced LAMP-based CRISPR/Cas12a method (ICB-LAMP-CRISPR/Cas12a) for the rapid visualization and detection of *Campylobacter jejuni*. It was captured by the ICB, heated to release the bacterial genomic DNA and used in the LAMP reaction, and the LAMP product was cleaved by CRISPR/Cas12a for detection. *Campylobacter jejuni* can be detected down to 8 CFU/mL. Furthermore, in addition to ICB capture, the method is performed in a closed pipeline to avoid aerosol contamination. The RT-LAMP-CRISPR-Cas13a method developed by Ortiz-Cartagena et al. (2022) does not require RNA extraction and can be used to detect SARS-CoV-2 viruses in nasopharyngeal samples with 100% specificity and 83% sensitivity (Figure 3).

### 3.2 RPA-CRISPR

RPA is a new nucleic acid thermostatic amplification technique developed by Piepenburg et al. in 2006 using protein recombination and repair machineries involved in cell DNA synthesis (Piepenburg et al., 2006). Since its establishment more than 10 years ago, RPA has been widely used in bacteria, fungi, parasites, viruses, drug resistance genes and other fields. Under ATP and PEG conditions, recombinase binds to primers to form a complex, and the DNA template of the complex searches for homologous sequences to start a strand-substitution reaction to form new DNA (Jaroenram and Owens, 2014; Daher et al., 2016; Jia et al., 2020; Tan et al., 2022). Compared with traditional PCR technology, this technology detection range is 37°C-42°C, the required sample concentration is low, can amplify as low as 1–10 DNA copies in 10 min, amplification of a variety of different targets, including RNA, miRNA, ssDNA, and dsDNA, RPA reactions can be detected by real-time fluorescence, gel electrophoresis, chemiluminescence and other methods (Munawar, 2022).

**Figure 4:**
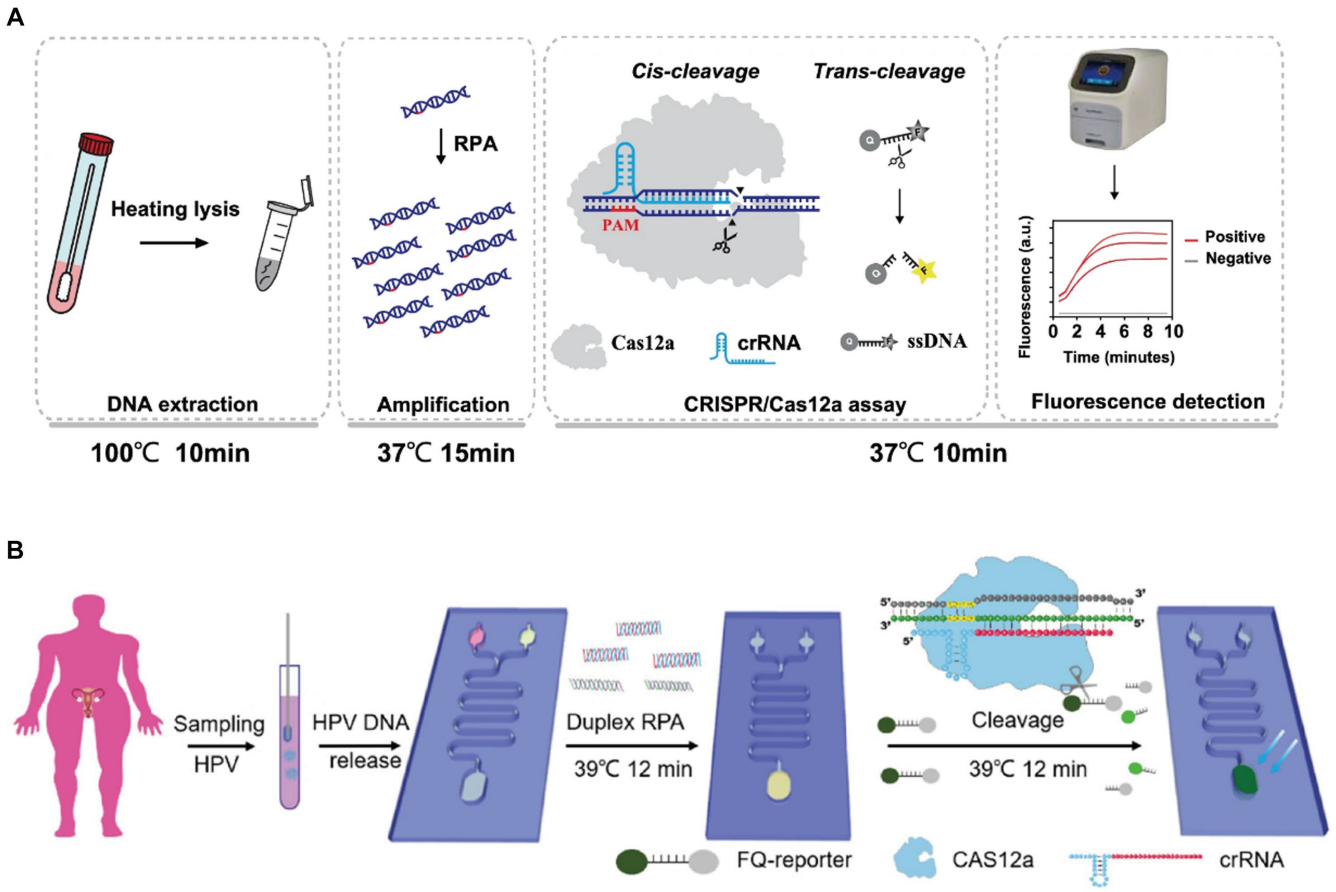
**(A)** Schematic diagram of the CRISPR-GBS assay (Yu et al., 2023); **(B)** detection of HPV in patient samples on CRISPR (Zhou et al., 2023).

Xiong et al. (2021) established a three-line lateral flow assay mediated by RT-RPA in combination with CRISPR/Cas9 for SARS-CoV-2 diagnosis. The analysis of 64 clinical samples showed that the negative predictive concordance was 100% and the positive predictive concordance was 97.14%. Liu et al. (2021) developed a food safety detection technology (RPA-Cas12a-FS) combining RPA and CRISPR-Cas12a for rapid detection of foodborne pathogens. Zhang et al. (2022) established CRISPR/Cas12a combined with RPA to detect *Aspergillus besseyi* with a LOD of 1 copy/μL, which was combined with a lateral flow strip assay to visualize the results. Yu et al. (2023) coarsely extracted DNA from vaginal or cervical swabs of pregnant women by heat lysis, then selected the highly conserved region of the cfb gene encoding the Christie-Atkins-Munch-Petersen (CAMP) factor as a target for detection, and enriched the target sequences by amplification using RPA, and finally utilised the CRISPR/Cas12a system for target-specific identification and signal release(Figure 4A). Our team Tian et al. (2021) combined the RPA-Cas12a technique with lateral flow immunoassay, thus realizing the rapid detection of *L. monocytogenes*. In addition, they developed a method combining digital droplet microfluidic chip technology to realize the simultaneous detection of four pathogenic bacteria with high sensitivity and specificity, which can reach 10 CFU/mL. The future development of this technology has great potential and may be applied to the rapid detection of foodborne pathogens, which is of great significance to ensure food safety. A multiplexed microfluidic platform developed by Zhou et al. (2023) combines RPA and CRISPR technology with the assistance of a heated membrane to enable rapid and cost-effective detection of multiple HPV subtypes while providing convenient temperature control for RPA and CRISPR analysis. The technology can detect HPV16 and HPV18 in less than 30 minutes with high specificity and sensitivity (Figure 4B).

### 3.3 SDA-CRISPR

SDA, or thermostatic amplification of DNA enzymes, is an *in vitro* DNA amplification technique based on the principle of DNA cleavage by restriction endonucleases at specific cleavage sites, as well as the extension of DNA polymerases at the cleavage sites, and the displacement of downstream DNA fragments in the thermostatic amplification technique (Walker et al., 1992). It also has good compatibility with various signal probes. These attributes render SDA an innovative signal amplification methodology with substantial potential for numerous types of biosensors, encompassing colorimetry, fluorescence, and chemiluminescence (Wang et al., 2022).

**Figure 5:**
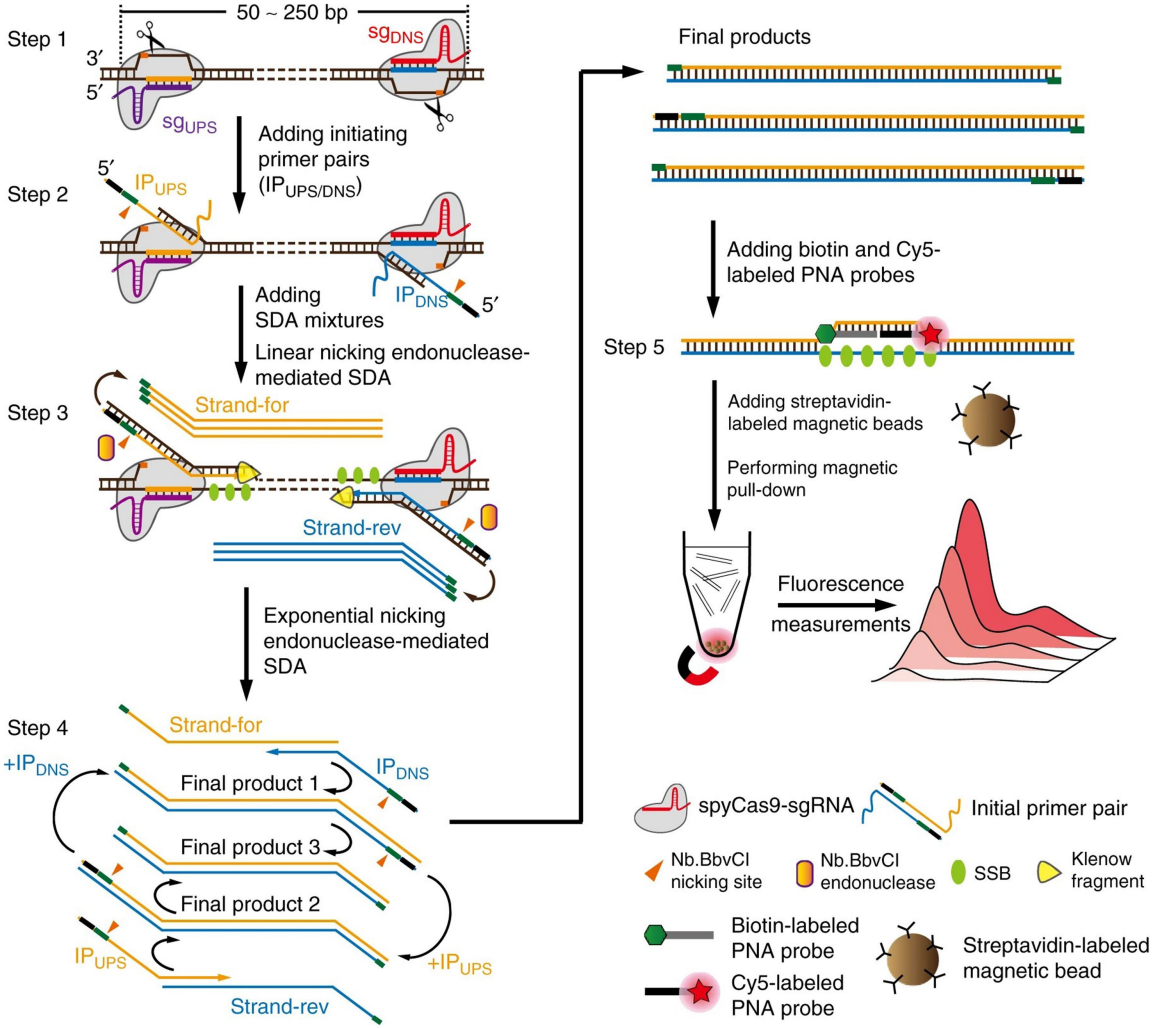
Schematic reaction mechanism of CRISDA (Zhou et al., 2018).

Combining real-time fluorescence detection with SDA provides ultrasensitive detection of ochratoxin A with a LOD of 0.01 ng/mL (Guo et al., 2022). Zhou et al. (2018) published a novel method named CRISPR-Cas9-triggered nucleic acid endonuclease-mediated signal-amplified DNA assay (hereinafter referred to as CRISDA), which employs the CRISPR-Cas9 system to amplify and detect double-stranded DNA (dsDNA) efficiently and sensitively. The proposed method employs the high sensitivity and accuracy of CRISPR for target DNA recognition, which is integrated with the powerful peptide nucleic acid (PNA) invasion-mediated endpoint detection to achieve sub-atomic level sensitivity and single-base specificity in even the most complex sample backgrounds (Figure 5). The researchers Wang et al. (2022) developed a colorimetric sensor platform that combines SDA with CRISPR/Cas12a for the detection of serum prostate-specific antigen (PSA). The platform is able to differentiate between blood samples were collected from prostate cancer patients, other types of cancer patients, and healthy individuals. PSA allows SDA to produce amplicons that are recognised by the CRISPR-Cas12a system and will mediate the cleavage of the trans ssDNA of the adjacent ligated DNA, thereby activating the gold nanoparticles (AuNPs), which signal the probe and provide a colourimetric readout with a LOD of 0.03 ng/mL, which reduces the background signal and improves the specificity of the assay. Gong et al. (2021) reported an SDA-assisted CRISPR-Cas12a method for the colorimetric analysis of viral nucleic acids. The Hepatitis B Virus DNA was used as a triggering target, activating the trans-cleavage activity of CRISPR-Cas12a and producing ssDNA. This ssDNA was then hybridized with template DNA and non-specifically cleaved on GOx-modified magnetic beads. The released GOx catalyzed the discoloration of the substrate solution and resulted in visualization of the results. Chi et al. (2022) combined SDA with Cas14 for the detection of circulating miRNAs, a biomarker of cholangiocarcinoma. miRNA was directly amplified by SDA without the need for reverse transcription, which reduced the risk of non-specific amplification, differentiated between miRNAs with similar sequences, and improved the sensitivity of the assay by detecting miRNAs as low as 680 fM within 1 h. Cas14a was efficiently activated by the single-stranded SDA amplicon with a 2.86-fold increase in sensitivity compared to the use of Cas12a (Figure 5).

### 3.4 HDA-CRISPR

HDA technology simulates *in vivo* DNA replication by using deconjugating enzymes to unravel the DNA double-stranded structure, primers to bind to the single-stranded target sequence, synthesize the new double-stranded DNA under the action of DNA polymerase, and repeat the amplification steps. The system requires only two primers (Vincent et al., 2004; Jeong et al., 2009; Barreda-García et al., 2018).

**Figure 6:**
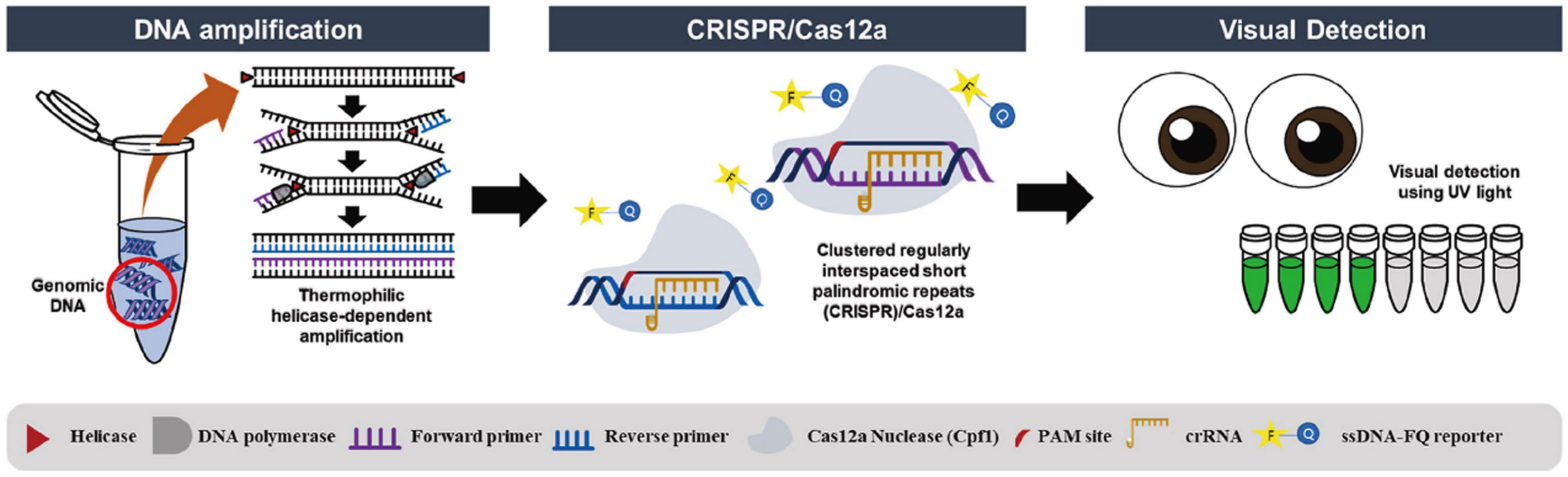
Schematic diagram of the detection of *E. coli O157:H7* based on HDA-CRISPR (Kim et al., 2023).

Thermophilic deconvolution enzyme-dependent amplification (tHDA) in combination with CRISPR/Cas12a specifically detects the virulence factor stx2 of *E. coli* O157:H7, eliminating false-positive results generated by primer dimers due to the binding of crRNA and Cas12a to the target, and detecting *E. coli* O157:H7 in salad mixtures as low as 10^3^ CFU/g (Kim et al., 2023; Figure 6).

### 3.5 RCA-CRISPR

RCA is a nucleic acid assay established to mimic the natural process of microbial circular DNA roll-over replication, acting on the circular DNA molecule (Nilsson et al., 1994), and small cyclic oligonucleotides act as templates for DNA or RNA polymerases to generate long and repetitive product strands (He et al., 2019). The two ends of the loop template are annealed with the ligating template and connected to make the template into a loop, after which the primers and the loop template are annealed and extended by DNA polymerase, and ultimately a long DNA single strand containing multiple repetitions of the template sequence can be obtained, with an amplification efficiency of up to 10^9^-fold (Deng and Gao, 2015).

**Figure 7:**
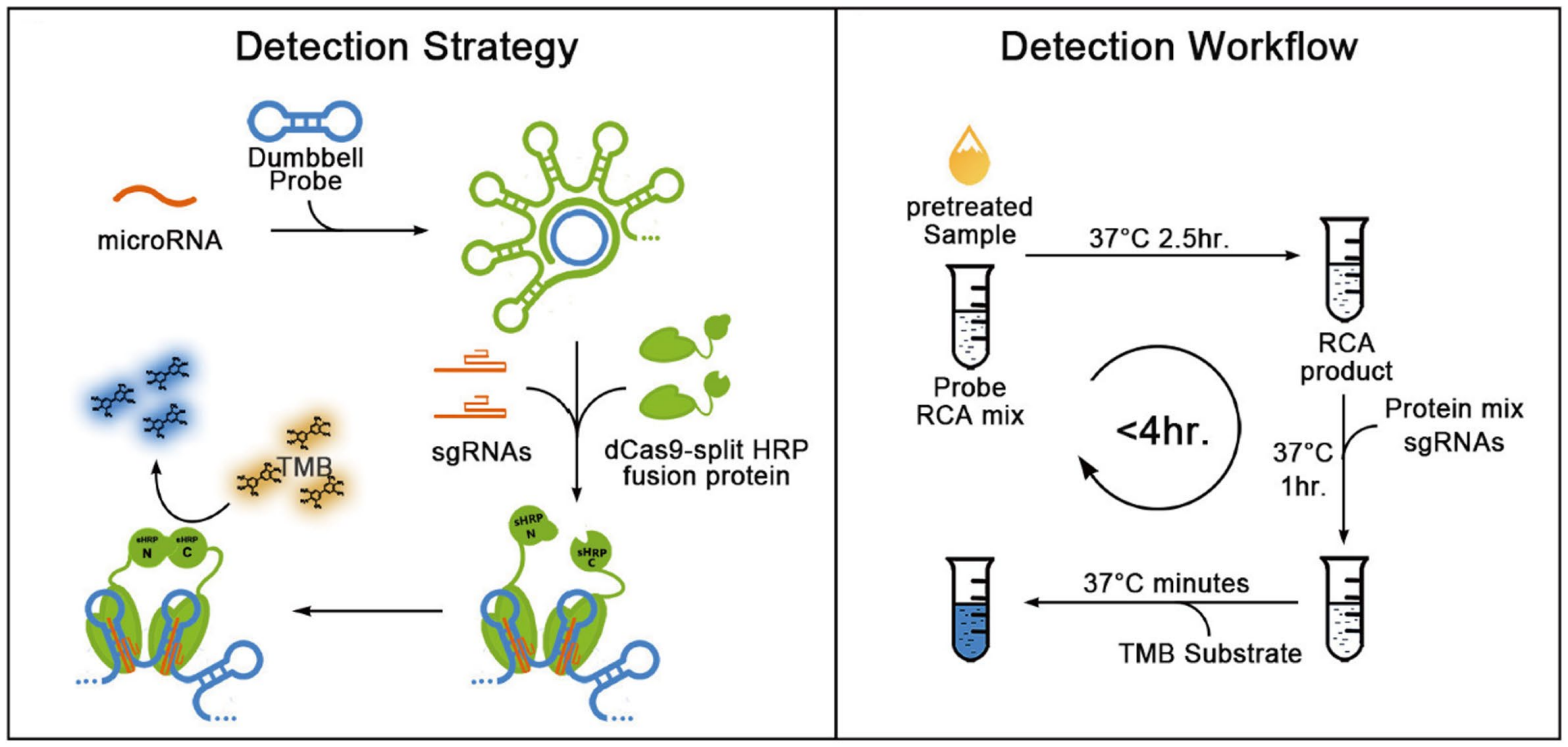
Schematic representation for the blueprint and detection workflow of the RCH method for detecting miRNAs (Qiu et al., 2018).

Qiu et al. (2018) investigated a novel RCA-CRISPR-split-HRP (horseradish peroxidase) method constructed from synthetic biological components. This technology enables the cost-effective and convenient detection of miRNAs with sensitivity as low as fM and single-base specificity. The substrate color changed from light yellow to blue after isothermal amplification by RCA for *in vitro* inactivation of Cas9 binding and splitting HRP activity assessment, indicating the presence of specific miRNAs. The whole process does not take more than 4 h, is easy to operate and does not require large equipment (Figure 7). Chen et al. (2023) combined branched-chain rolling ring amplification (BRCA) and CRISPR-Cas12a (BRCA-Cas) to establish a detection platform for colorectal cancer-related circulating non-coding RNA, which realized the detection of different circulating RNA in serum. Abnous et al. (2021) combined CRISPR-Cas12a, RCA and AuNPs to introduce an aptamer sensor for highly sensitive detection of aflatoxin and the color of the sample changed from yellow to colorless. The addition of T4 DNA ligase and phi29 DNA polymerase in the presence of the target results in the inactivation of the CRISPR-Cas12a gene editing system and the formation of larger single-stranded DNA structures on the surface of AuNPs. Qing et al. (2021) introduced an immobilization-free electrochemical biosensing platform into the CRISPR/Cas system, which can be used to accurately detect nucleic acids and small molecules associated with diseases, where RCA converts and amplifies the target recognition sequences and is used to activate CRISPR/Cas12a activity. Universal blocker probe (BP) can be cleaved by activated Cas12a, which is not the case for unactivated Cas12a. This electrochemical biosensor combines an RCA-CRISPR/Cas12a platform with a rGO/GCE electrode to detect dsDNA, yielding a cost-effective and versatile tool for clinical diagnostics.

### 3.6 HCR – CRISPR

**Figure 8:**
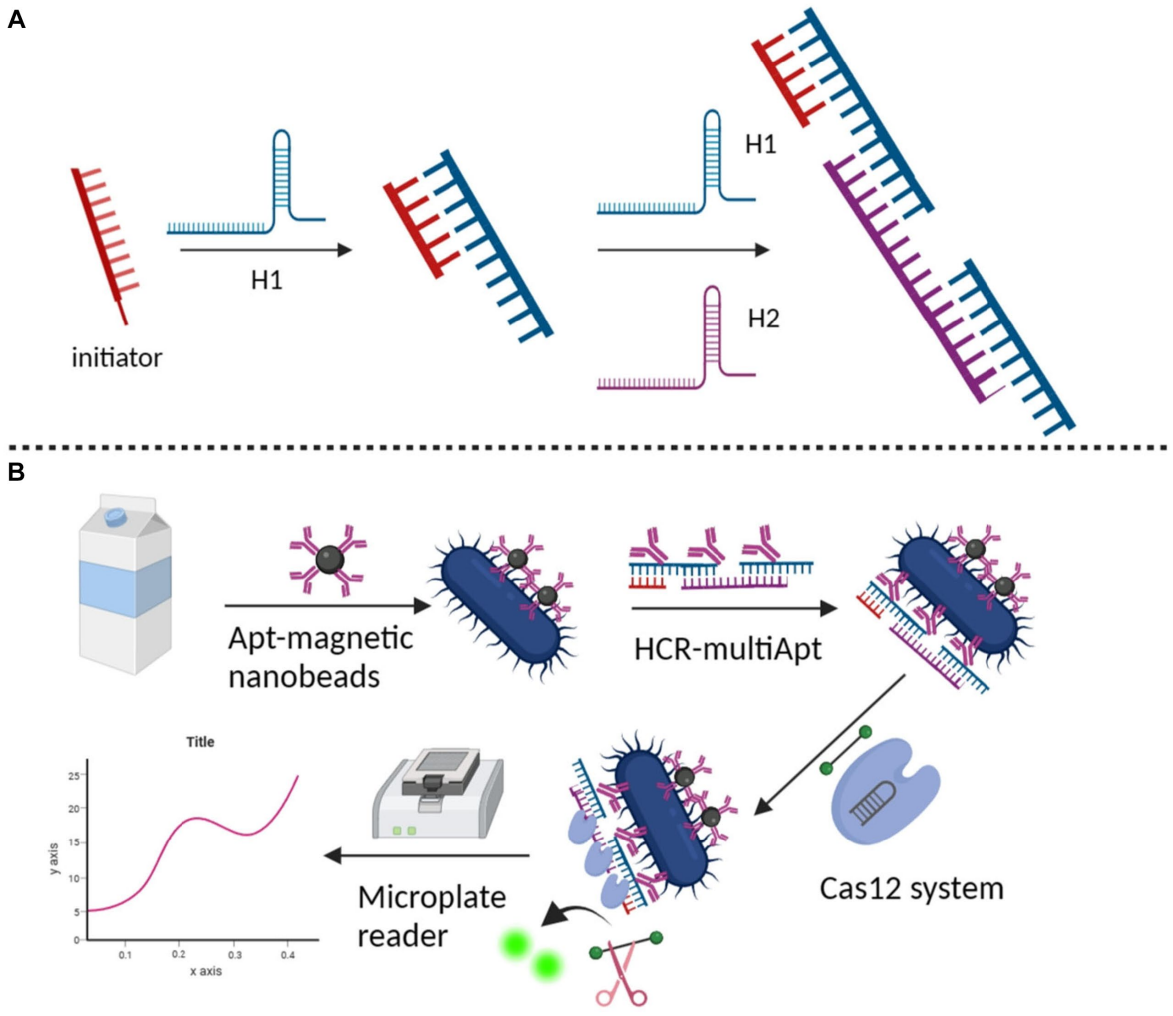
**(A)** Fundamental working principle of HCR (Zhang et al., 2020); **(B)** detection of HPV in patient samples on CRISPR (Qiao et al., 2023).

Dirks and Pierce (2004) introduced the HCR, a type of isothermal and enzyme-free nucleic acid amplification technology, in 2004. HCR is gaining popularity in biotech, forensics, and infectious disease detection. A single-stranded DNA initiator triggers an alternating hybridisation event between two hairpins forming a double helix polymer (Figure 8A; Bi et al., 2017; Zhang et al., 2020; Li et al., 2022). HCR with its unique properties of isothermal, enzyme-free, and high amplification efficiency is widely used in biosensing and biomedical sectors due to its excellent analytical abilities and wide application potential (Zhou et al., 2021).

CRISPR/Cas systems have ignited increasing attention in accurate and sensitive nucleic acids detection. Ke et al. (2022) proposed a new CRISPR/Cas12a-based chemiluminescence enhancement biosensor for nucleic acid detection. With a remarkable sensitivity, it is capable of detecting synthetic DNA targets at a detection limit of 3 pM, and can also single-copy detect plasmids. This study demonstrated the high accuracy of HPV testing, achieving a sensitivity of 88.89% and specificity of 100% in practical application, respectively, showing its superior accuracy. Xing et al. (2020) based on the dual amplification of HCR and CRISPR-Cas12a, we developed the apta-HCR-CRISPR assay for the direct, highly sensitive detection of tumor-derived extracellular vesicle (TEV) protein. HCR amplified TEV protein-targeted aptamer with long-repeated sequences and multiple barcodes, enhancing detection sensitivity and specificity. CRISPR-Cas12a boosts fluorescence signals for highly sensitive quantification of TEV proteins in clinical samples. Qiao et al. (2023) established an amplification free detection method for Salmonella by magnetic separation of dual-function HCR scaffold polyvalent aptamers with CRISPR/Cas12a activity coupling. In the detection system, the dual-function HCR scaffold polyvalent aptamers with high binding affinity and specificity were first prepared by assembling several *Salmonella*-specific aptamers on the long HCR products. In addition to the enhanced affinity, HCR-Multiapt also contains many repetitive CRISPR-targeting DNA units in its HCR scaffold, which may trigger the trans-cutting activity of Cas12a. In the presence of the target bacteria, HCR-scaffold polyvalents can efficiently attach to the bacterial surface and amplify the bacterial signal into a CRISPR/Cas12a based fluorescent readout. The detection system can perform ultra-sensitive detection of *Salmonella* in the linear range of 10^0^ ~ 10^7^ cfu/mL with a detection limit of 2 cfu/mL. This method, which possesses high sensitivity, can be effectively utilized for clinical diagnosis (Figure 8B). With the use of CRISPR-Cas13a and enzyme-free nucleic acid amplification. Yang et al. (2021) reported the development and validation of a HCR coupled CRISPR-Cas13a-based assay (Cas-HCR) for detection of SARS-CoV-2. The detection performance of Cas-HCR assay was demonstrated on a home-made optical-fiber evanescent wave fluorescence biosensor capable of collecting the fluorescence excited within an exponentially decaying evanescent field around the optical fiber.

### 3.7 Other isothermal amplification techniques – CRISPR

In addition to the above widely used isothermal amplification technologies, there are many isothermal technologies that are being deeply explored and improved. Enzymatic Recombinase Amplification (ERA) is a domestic independent research and development amplification technology, the reaction relies on recombinase derived from low-temperature bacteriophages, can replace amino acids at specific sites, has good amplification speed and specificity, combines ERA with Cas12a to achieve highly sensitive detection of porcine circovirus (Zhang et al., 2021). Real-time fluorescence nucleic acid isothermal amplification detection (SAT) is a combination of nucleic acid isothermal amplification and real-time fluorescence detection, which can design specific primers for the target nucleic acid of pathogens, and can rapidly amplify and detect *Mycoplasma pneumoniae* with high sensitivity and specificity (Barreda-García et al., 2018; Li et al., 2019). NASBA technology amplifies target RNA under thermostatic conditions by using single-stranded RNA as a template to mimic the replication mechanism of retroviruses *in vivo* by reverse transcriptase, RNase H and T7 RNA polymerase, and forward and reverse primers (Compton, 1991), and T7RNA polymerase recognizes the DNA promoter sequence and transcribes it into single-stranded RNA for the next cycle (Hønsvall and Robertson, 2017; Ju et al., 2021). Wang et al. (2021) used NASBA technology to diagnose clinical samples from 25 patients with cryptococcal disease with LOD of 10 CFU/mL. Pardee et al. (2016) designed a paper-based sensor for rapid detection of Zika virus and binding to CRISPR/Cas9 to identify virus strains, a platform that can directly detect 2.8 fM Zika virus RNA from infected rhesus monkey serum. This system displays the results through color changes, and can be judged by the naked eye, reducing the cost of use and improving the range of use. The novel exonuclease assisted isothermal nucleic acid amplification (Exo-NAT) technology developed by Ye et al. (2018) was analyzed by using full-length Bst DNA polymerase combined with melting curve, which had ultra-high specificity and good detection limits in both singleplex and multiplex detection, and showed *Rotavirus A* and *Rotavirus A* in 42 clinical samples. Astrovirus and adenovirus have been validated with up to 100% specificity and sensitivity. The Hairpins Mediated Amplification (HMA) technique developed by Hongbo et al. (2022) combines LFD to detect specific amplification products and reduce the signal of non-specific oligonucleotide hybridization, thereby making it more specific. HMA was validated by detecting IS6110 fragments of Mtb, combined with LFD to visualize the results. Isothermal Exponential Amplification Reaction (EXPAR) technology relies on the synergy of nickease and DNA polymerase with strand displacement function to achieve exponential amplification of targets in buffer systems containing dNTP, primers (in most cases, targets to be measured), and amplification templates (Mok et al., 2016; Reid et al., 2018). Huang et al. (2018) created a groundbreaking CRISPR/Cas9-based isothermal exponential amplification reaction strategy for rapid and site-specific nucleic acid detection. CRISPR/Cas9 with exponential amplification generates multiple DNA replicas that are detected by real-time fluorescence monitoring. The method is characterized by specificity and rapid amplification kinetics.

Among these, isothermal NAATs have garnered interest due to their operation at a constant temperature (thus requiring minimal laboratory setup) and cost-effectiveness (Dueñas et al., 2022), the combination of isothermal NAATs and CRISPR/Cas technology has been successfully applied in the rapid detection of various pathogens, which has the advantages of low detection cost, high precision, fast quantification, and on-site rapid detection, and has a high potential for popularization and application. Compared to PCR, isothermal NAATs have reduced temperature requirements and assay costs, and different isothermal NAATs combined with CRISPR/Cas systems have different results. The programmable features of CRISPR-Cas12a, accurate target identification and indiscriminate cutting characteristics offer great promise for building fluorescence and colorimetric sensing platforms, especially since the CRISPR-Cas12a system has good compatibility with SDA and other warm signal amplification technologies (Wang et al., 2022). Isothermal amplification, as a novel technology, has many advantages but also faces many challenges, such as false positive signals generated by LAMP non-specific amplification and the complexity of primer design are still widely used technical challenges (Zhang et al., 2021). Compared to other isothermal techniques, RPA is limited by the concentration of DNA, too high a concentration will inhibit the reaction, the amplification process is easily contaminated and the purification process of RPA products is difficult. NASBA amplification has many steps, the reaction system is more complex, not easy to develop, the reaction system needs to be added to the enzyme reaction solution, and the cost is high (Debayan et al., 2023). The HDA technique requires 2 to 3 reaction temperatures, and the unlooped lock-in probe and template DNA or RNA that is not bound to the lock-in probe during the RCA process can also generate signals, which may reduce the sensitivity of the assay, and continuous improvement is still needed. Correctly and efficiently amplifying target sequences at appropriate temperatures and being able to accurately detect sequences and distinguish signals without interference is one of the major problems facing isothermal amplification technology today. Despite the simplicity, low cost, and high accuracy of advanced CRISPR-Cas-based biosensors, the synergistic use of CRISPR-Cas-based dual signal amplification systems for rapid diagnosis of pathogens is still rare (Tables 1, 2).

**Table 1:** Comparison of the characteristics of different isothermal amplification techniques.

Number	Technology name	Primer number	Temperature (°C)	Time (min)	Enzyme species	Template type	Efficiency	Detection method
1	LAMP	4–6	60–65	20–60	DNA polymerase	DNA/RNA	10^9^–10^10^	Gel electrophoresis, turbidimetry
2	RPA	2	37–42	15–60	Recombinase, DNA polymerase	DNA/RNA	/	Fluorescence, gel electrophoresis, chemiluminescence
3	NASBA	2	40–55	60–120	Reverse transcriptase, RNaseH	RNA	10^9^–10^12^	Chemiluminescence
4	HDA	2	65	75–90	Helicase, DNA polymerase	DNA	10^7^	Fluorescence
5	RCA	1	37	90–180	DNA polymerase, ligase	DNA/RNA	10^9^	Gel electrophoresis
6	ERA	4	37–42	15–30	Recombinase, DNApolymerase	DNA	10^12^	Gel electrophoresis, fluorescence
7	SAT	2	42	15–30	Reverse transcriptase, T7RNA	RNA	10^9^	Fluorescence
8	IMSA	6	60–65	60–90	DNA polymerase	DNA/RNA	/	Gel electrophoresis, color determination, turbidity, fluorescence
9	Exo-Nat	8	60–68	90	DNA polymerase	DNA/RNA	/	/
10	SDA	4	65	30–60	DNA polymerase, NEase	DNA	10^8^	Colorimetry, fluorescence, chemiluminescence
11	HMA	2	63	60–180	DNA polymerase	DNA/RNA	/	/
12	EXPAR	/	55	30	NEase, DNA polymerase	DNA/RNA	/	/
13	HCR	4	/	30	/	DNA/RNA	/	Colorimetry, fluorescence, chemiluminescence

**Table 2:** Comparison of CRISPR/Cas detection methods based on nucleic acid amplification.

Amplification technology	Type	Detect target	LOD	References
PCR	Cas9	Tumor	/	Meng et al. (2023)
		Invasive ductal carcinoma	/	Rakshit et al. (2022)
	Cas12	*Salmonella*	5%	Yang et al. (2022)
		GM crops	<60 copies	Wang et al. (2023)
	Cas13	*S. aureus*	1 CFU/mL	Zhou et al. (2020)
		Carbapenem-resistant *Klebsiella pneumoniae*	1 copy/μL	Cao et al. (2023)
		Hepatitis B virus (HBV), Hepatitis D virus (HDV)	1 copy/μL	Zhang et al. (2022) and Tian et al. (2023)
LAMP	Cas9	*Salmonella*, five *Neisseria meningitidis* serotypes, *Zika* virus	/	Bao et al. (2020)
	Cas12	*Salmonella*	1.22 × 10^0^ CFU/mL	Lee and Oh (2023)
		SARS-CoV-2	/	Wang et al. (2021) and Cao et al. (2022)
		*Campylobacter jejuni*	8 CFU/mL	Li et al. (2022)
	Cas13	SARS-CoV-2	/	Ortiz-Cartagena et al. (2022)
RPA	Cas9	SARS-CoV-2	/	Xiong et al. (2021) and van der Veer et al. (2023)
		Single base mutations	0.2 fM	Zhou et al. (2022)
	Cas12	Foodborne pathogens	/	Liu et al. (2021)
		*Aspergillus besseyi*	/	Zhang et al. (2022)
		*Streptococcus agalactiae*	5 copies/μL	Yu et al. (2023)
		*L. monocytogenes*	10 CFU/mL	Tian et al. (2021)
		HPV16 and HPV18	/	Zhou et al. (2023)
SDA	Cas9	dsDNA	/	Zhou et al. (2018)
		*E. coli* O157:H7	4.0 × 10^1^ CFU/mL	Sun et al. (2020)
	Cas12	PSA	0.03 ng/mL	Wang et al. (2022)
		HBV	41.8 fM	Gong et al. (2021)
		Cadmium ion	60 pM	Ma et al. (2023)
		miRNA	6.28 pM	Feng et al. (2023), Wang et al. (2022), and Zhu et al. (2023)
		SARS-CoV-2	2.7 × 10^2^ copies/mL	Shi et al. (2023) and Liu et al. (2023)
	Cas14	miRNA	680 fM	Chi et al. (2022)
HDA	Cas12	*E. coli* O157:H7	10^3^ CFU/g	Kim et al. (2023)
RCA	Cas9	miRNA	fM level	Qiu et al. (2018)
	Cas12	miRNA, Parvovirus B19 DNA, Adenosine-5′-triphosphate	0.83aM, 0.52aM, 0.46 pM	Qing et al. (2021)
		ncRNAs	/	Chen et al. (2023)
		AFM1	0.05 ng/L	Abnous et al. (2021)
HCR	Cas12	Circulating tumor DNA	5.43 fM	Li et al. (2023)
		*Salmonella*	2 cfu/mL	Qiao et al. (2023)
		Alpha-fetoprotein	0.170 ng/mL	Liu et al. (2022)
		HPV	/	Ke et al. (2022)
		TEV	10^2^ particles/μL	Xing et al. (2020)
	Cas13	SARS-CoV-2	/	Yang et al. (2021)

## 4 Sample pretreatment and portable detection devices

Nucleic acids, being essential components of all living organisms, provide a wealth of information regarding biological and pathological aspects of life (Zhang et al., 2022). The impact of sample pretreatment on nucleic acid amplification technology is very important, and it can directly affect the efficiency and accuracy of the amplification reaction. DNA or RNA extraction from the sample is the first step in nucleic acid amplification, and the quality and purity of the extraction is critical to the amplification reaction. Low-quality DNA or RNA may result in inefficient amplification, spurious products, or failure. Therefore, selecting the appropriate extraction method and kit and following best practices for the extraction steps can ensure a high quality nucleic acid sample (He et al., 2017; Mullegama et al., 2019). Some inhibitory substances may be present in the sample (Lantz et al., 1996), such as hemoglobin, anticoagulants, and polyketides in blood. These inhibitory substances can affect the efficiency and accuracy of the amplification reaction. Therefore, it is necessary to use appropriate methods to remove these inhibitory substances during sample pretreatment, such as using special extraction kits, purification columns, or other removal methods. The impact of portable devices on nucleic acid amplification technology has been significant, allowing for the amplification of nucleic acids outside of the laboratory, providing easier, faster and more flexible applications (Wang et al., 2020; Chen et al., 2023).

## 5 Discussion

Pathogens can cause serious diseases in humans and animals, and detection technology based on nucleic acid amplification plays an important role in pathogen detection. PCR is still the most mature technology in detection, and isothermal nucleic acid amplification technology is becoming more mature in microbial detection, which can compensate for PCR’s dependence on application scenarios and instrumentation, and has the characteristics of rapid, sensitive and convenient detection, and is especially suitable for rapid on-site detection in remote areas (Gill and Ghaemi, 2008). It is the main direction of future development in the field of pathogen nucleic acid detection (Deng and Gao, 2015; Jiang et al., 2023). However, compared with traditional PCR, primer design in isothermal nucleic acid amplification is complicated and difficult, lacks appropriate design software, requires screening and optimization of reasonable primers (Mayboroda et al., 2018), and is more prone to false-positive results due to aerosol contamination or non-specific amplification, and there is still a certain gap between detection sensitivity and qPCR. Methods that enable rapid and on-site detection of pathogenic bacteria are a prerequisite for public health assurance, medical diagnosis, ensuring food safety and security, and research. Many current bacterial detection technologies are inconvenient and time-consuming, making them unsuitable for on-site detection. New technologies based on the CRISPR/Cas system have the potential to fill the existing detection gap (Chakraborty et al., 2022). CRISPR-based sensors for the detection of pathogens, proteins, miRNAs, etc., have a wide range of applications (Wang et al., 2022; Zhang et al., 2023). There are risks associated with CRISPR technology: it may lead to off-target effects (Cheng et al., 2020), i.e., the editing process may accidentally alter DNA sequences in non-target regions of the genome, which may lead to unintended consequences, such as genetic mutations or cytotoxicity. The immune effect triggered by the Cas protein itself is also one of the shortcomings (Sun et al., 2015). Unselected ssDNA cleavage activity of cas12a impairs targeted host cells (Paul and Montoya, 2020). Although there are still some problems with CRISPR/Cas9 gene editing technology, researchers have been committed to finding solutions, and through a series of measures such as modification of Cas protein, use of Cas direct homologous enzyme, and chemical assistance, CRISPR/Cas9 gene editing technology has improved its ability to accurately repair broken DNA, reduced its off-target rate, and weakened the target site restriction caused by PAM. target site restriction, controlling the occurrence of mosaicism to a certain extent. Furthermore, we are aware of the limitations of our CRISPR-Cas13a based assay and recognize the enhancements that would be required prior to its potential utilization in resource-limited settings as a clinical POC test. While researchers are looking for ways to make up for the shortcomings, they are also expanding the application areas of this technology (Knott and Doudna, 2018). The combination of isothermal nucleic acid amplification and CRISPR/Cas system can realize the secondary amplification of the detection signal and effectively improve the detection sensitivity, which has been applied in some applications in rapid detection, but it is mainly for a single pathogen, and needs to be combined with the multiplex nucleic acid amplification technology to improve the detection throughput and realize the multi-targeted joint detection. CRISPR/Cas technology has a very promising future in nucleic acid amplification technology. It can provide higher specificity, target selection ability, multiple amplification ability, while combining with gene editing and modification to realize real-time monitoring and detection, etc. With the continuous development and improvement of the technology, CRISPR/Cas technology is expected to bring more innovations and applications in nucleic acid amplification technology.

In conclusion, the rapid emergence of CRISPR-Cas technology for genome manipulation has been revolutionary for the life sciences. Despite the broad spectrum of applications of this technology, CRISPR molecular tools available at present face a range of limitations and challenges: e.g., reliance on DNA repair mechanisms, safety and ethical issues all need to be addressed with attention. In the future research, nucleic acid amplification technology still needs to be integrated, high-throughput, multiplexed and convenient as the research objectives, from the various technical difficulties, optimize the pre-treatment method, primer design method, product detection method and so on, in order to improve the efficiency of the detection; nucleic acid amplification technology has an important application prospect in the field of nucleic acid analysis. With the continuous development and innovation of the technology, it is believed that nucleic acid amplification technology will play a greater role in biomedical research, disease diagnosis and environmental monitoring, and bring more new breakthroughs and progress in the field of life sciences. With the continuous development and innovation of the technology, it is believed that nucleic acid amplification technology will play a greater role in biomedical research, disease diagnosis and environmental monitoring, and bring more new breakthroughs and progress in the field of life sciences.

## Author contributions

DZ: Writing – original draft, Writing – review & editing. JJ: Writing – review & editing. TM: Writing – review & editing.
